# A research report on a novel typological study of the Chinese metaphorical and metonymic idioms

**DOI:** 10.3389/fpsyg.2024.1321778

**Published:** 2024-03-04

**Authors:** Yangyang Xi

**Affiliations:** School of Chinese Language and Literature, Wuhan University, Wuhan, China

**Keywords:** Chinese metaphorical and metonymic idioms, Spreading-Activation Model, novel classification, continuum of cognitive difficulty, teaching Chinese as a foreign language

## Abstract

This report shows a novel classification of the Chinese metaphorical and metonymic idioms based on the Spreading-Activation Model (SA Model). Identified first are three features of the source domain within the Chinese metaphorical and metonymic idioms: (1) the relationship between the source and target domains, (2) the number of source domains within idioms, and (3) the inherent characteristics of the source domain. Then drawing on the SA Model, this report arrives at a novel classification of the Chinese metaphorical and metonymic idioms into four categories: (1) Directional activation Idioms, (2) Cooperative activation Idioms, (3) Spreading activation Idioms, and (4) Superimposed activation Idioms. Discussion is made on each category and a continuum of cognitive difficulty is established with respect to understanding different categories. This classification is of great help within the realm of teaching Chinese as a foreign language.

## 1 Introduction

In the Chinese language system, idioms constitute a highly significant component owing to their expressiveness and wide use. In the past two decades, research on Chinese idioms primarily covers the disciplines of linguistics and psychology, showcasing a trend of integrating theory with empirical research. Psychology employs empirical methods, encompassing techniques such as priming effects, eye-tracking, event-related potentials, and virtual reality technology, continuously introducing innovative experimental approaches (Zhong, 1998; Su, 2013; Ke, 2021; Wang and Peng, 2022). In contrast, linguistic studies reveal a shift from “theory” toward “theory + empirical,” firmly establishing idioms as the central focus of investigation.

The breakthroughs in idiom research come from the introduction of activation theory and the utilization of new technologies as well as the development of cognitive linguistics. Activation theory comprises two processes: constructionist and reductionist. Constructionist focuses on internal relationships within idioms, while reductionist emphasizes the correlation between literal and metaphorical meanings (Zhang et al., 2012; Wang, 2020).

Historically, discussions on idioms have predominantly occurred at the linguistic level. With the rise of cognitive linguistics and the development of cognitive metaphor theories, scholars are increasingly able to elucidate idiom content from fresh perspectives.

Within the realm of teaching Chinese as a foreign language (TCFL), idiom instruction is anything but an easy task in that they are numerous and significantly metaphorical or metonymic in nature. Given that the Chinese idioms are mostly metaphorical and metonymic, great importance is attached to the study of the Chinese metaphorical and metonymic idioms (CM&MIs).

With this context, this report revolves around the core theme of “An Attempted Application of Activation and conceptual metaphor theories to formulating a novel classification of the CM&MIs and providing adequate interpretation for them to facilitate the understanding and instruction of the Chinese idioms in the realm of TCFL. In addition, a continuum is established concerning the difficulty in understanding the CM&MIs.

More specifically, this report proposes a new perspective on idiom research, using three characteristics of the source domain as entry points, connecting with the Spreading-Activation Model, breaking through existing theoretical frameworks, and proposing a new idiom classification model, offering novel insights and methodologies for idiom research and instruction (Chen, 2010; Zhang et al., 2012; Wen, 2017; Wei, 2019).

## 2 Methodology

The idioms employed in this report are taken from the six volumes of *Developing Chinese*. They are processed through three approaches for the description and interpretation of the CM&MIs and one method for specifying the grading of them in terms of cognitive difficulty they require.

### 2.1 Three approaches and their application to the novel typology of the CM&MIs

#### 2.1.1 Conceptual metaphor and the integration of the CM&MIs

**Figure 1:**
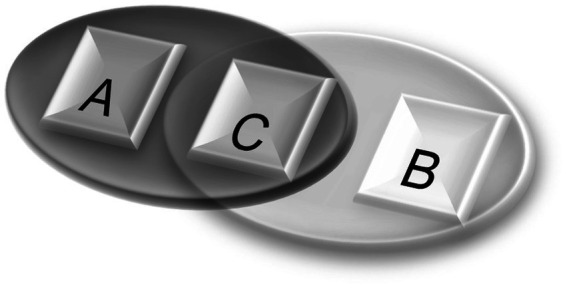
Integration of metonymy and metaphor categories of idioms.

Different from the traditional notion of metaphor and metonymy as figures of speech, metaphor and metonymy are conceptual in cognitive linguistics, both involving mapping from source domain onto target domain. Metaphor is based on the mapping between the source and target domains, two different domains, while metonymy is based on the mapping from the source domain onto the target domain within a domain. The former deals with analogical relationships, and the latter involves referential relationships (Lakoff and Johnson, 1980; Ungerer and Schmid, 1996; Croft and Cruse, 2004). Both metaphor and metonymy, however, have a mechanism in common, namely, mapping. Therefore, this report combines the CM&MIs into a unified category. This integration resolves certain issues related to the difficulty of determining whether an idiom belongs to metonymy or metaphor, as well as situations where an idiom can belong to both categories simultaneously (see Figure 1).

There are three relevant concepts concerning the CM&MIs: the source domain, the target domain, and the linking typically being overt in metaphor and covert in metonymy between the former two concepts. Among these, the source domain is a mandatory element within the idioms. Therefore, this report employs the source domain as the starting point and focuses on the three characteristics of source domains within idioms: (1) the relationship between source and target domains, (2) the number of source domains within idioms, and (3) the specific attributes of source domains.

#### 2.1.2 Compositionality and the CM&MIs

In semantic studies, the concept of “compositionality” is related to the organization of meaning. For a linguistic unit composed of several components, its inherent meaning depends on the semantics of these components and how they are combined. Idioms, as members of fixed phrases, often have meanings that are difficult to figure out using ordinary word formation rules, and they can be viewed as language structures consisting of four Chinese characters generally with different degrees of compositionality. These four characters can individually represent conceptual components, such as in “衣食住行” where each character represents an independent conceptual component, semantically distinct. They can also combine in pairs to form conceptual components, as in “鸡毛蒜皮,” where “鸡” and “毛” combine to form “鸡毛,” and “蒜” and “皮” combine to form “蒜皮.”

According to Langlotz’s (2006) model, there exist syntagmatic integration relationships between the components, while there are compositional relationships between the composite structure and the components. The compositionality hypothesis in idiom processing posits that there exists a metaphorical relationship, rather than an arbitrary one, between the overall meaning of idioms and the constituent elements of idioms (Gibbs, 1992; Xiao, 2000; Glucksberg, 2001; Zhang, 2003, 2008;Yang and Zhang, 2007).

#### 2.1.3 The Spreading-Activation Model (the SA Model)

**Figure 2:**
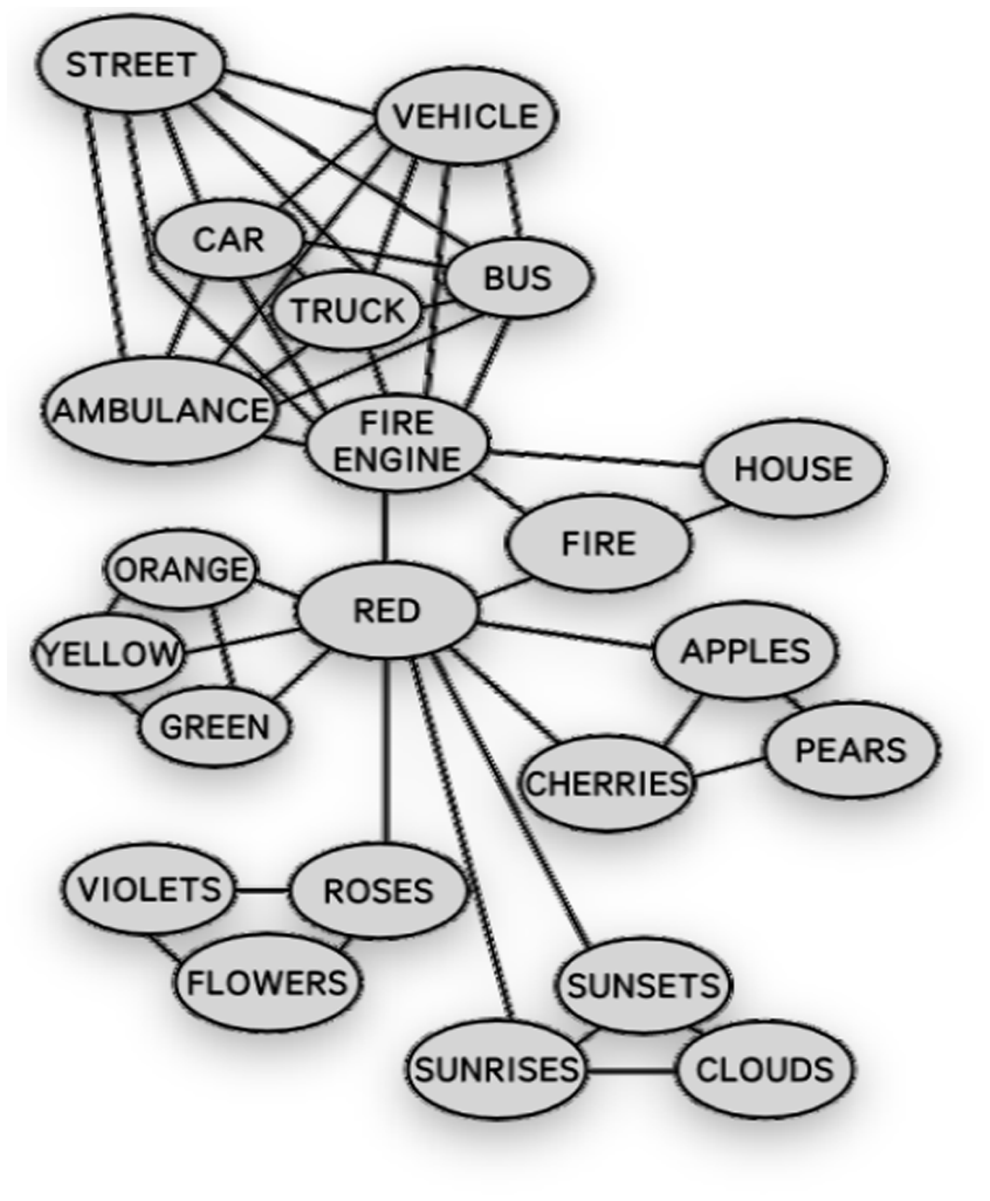
The Spreading-Activation Model.

The Spreading-Activation Model was proposed by Collins and Loftus (1975). Spreading-Activation is the recursive spreading process of initial stimulation. This model posits that when a concept is stimulated, it initiates an activation process, and its effects spread in all directions, propagating to other connected concepts. However, the energy of activation diminishes with increasing distance, meaning that closely related concepts are more likely to be activated than those farther away. From the operative perspective, the SA Model functions as an organizational method of human memory. It asserts that memory organization resembles a complex network of connections, where specific memories diffuse among relevant concepts. In the SA Model, the strength of connections between concepts is represented by the links between nodes. The shorter the link, the stronger the connection, and conversely, the longer the link, the weaker the connection (see Figure 2).

### 2.2 Continuous grading analysis method

These four distinct categories of idioms are characterized each by unique activation patterns that correspond to varying degrees of cognitive difficulty. In Section 3.3, we assess the relative cognitive difficulty of the CM&MIs in terms of three key factors: activation number, presence of assistance, and the nature of assistance. Correspondingly, we establish a continuous gradient of cognitive difficulty and recognize that the complexity of idiom comprehension is contingent upon the intricacies of their activation patterns. To rephrase our objective, evaluating the cognitive difficulty of four categories of idioms is equivalent to determining the complexity of their respective activation patterns. For details, see Section 3.3 Cognitive Difficulty Result of Four Categories of the CM&MIs.

## 3 Results and discussion

Drawing on the four approaches listed in the second part to attempt a novel classification gives rise to the four categories of the CM&MIs below, and a detailed discussion is made on each of them.

### 3.1 The four categories of the CM&MIs

#### 3.1.1 Directional activation idioms

Directional activation idioms, where both the source and target domains co-occur, manifest a distinctive pattern termed directional activation. Taking the idiom “暴跳如雷” (bàotiào rú léi) from *Developing Chinese* as an example, the source domain “雷” (léi), signifying “thunder,” intersects with the target domain “暴跳” (bàotiào), meaning “jumping furiously.” This co-occurrence creates a Spreading-Activation map in brain. Thunder, perceived through sound, embodies loudness; tangibly, it associates with natural phenomena like rain, snow, storms, and hail; mystically, it signifies the wrath of celestial deities, foretelling ominous signs. However, “暴跳” (jumping furiously) directs the meaning of “雷” (thunder) toward a furious anger, akin to a celestial deity. Thus, the unidirectional and fixed activation from the source domain to the target domain can be likened to an arrow, focusing the spreading energy and highlighting the idiomatic essence.

These directional activation idioms are not rare, constituting 18.1% of idioms in *Developing Chinese* (56 in total). Examples include “受宠若惊” (shòu chǒng ruò jīng, flattered but also anxious), “聪明绝顶” (cōngmíng jué dǐng, extremely clever), “小心翼翼” (xiǎoxīn yìyì, extremely cautious), “门庭若市” (méntíng ruòshì, thronging with visitors), and “含情脉脉” (hánqíng mò mò, with affectionate looks). In “受宠若惊,” the source domain “惊” (jīng, anxious) guides the target domain “受宠” (shòu chǒng, being flattered), conveying the idea of being pleased by favor but also feeling uneasy. Similarly, “聪明绝顶” uses “绝顶” (jué dǐng, utmost) to direct to “聪明” (cōngmíng, clever), describing extreme intelligence beyond comparison.

#### 3.1.2 Cooperative activation idioms

Cooperative Activation Idioms, belonging to the multiple-source category, exhibit a distinct activation pattern. These idioms possess two notable features: only the source domain is explicitly present, and there are two or more source domains simultaneously at play. For instance, the idiom “鸡毛蒜皮” (jīmáo suànpí) intertwines “鸡毛” (jīmáo) and “蒜皮” (suànpí). From a tangible perspective, “鸡毛” signifies a serious or meticulous attitude, typically indicating determination and persistence in achieving a goal. Yet, in the idiom “拿着鸡毛当令箭” (názhe jīmáo dāng lìngjiàn), “鸡毛” refers to trivial or generalized words. In this cooperative activation, the two source domains intersect in a specific overlapping area, cooperatively generating the meaning of trivial and unimportant matters. Examples of idioms with four source domains include “衣食住行” (yī shí zhù xíng), where “衣” (yī, clothing), “食” (shí, food), “住” (zhù, shelter), and “行” (xíng, transportation) collectively form independent semantic networks. However, within this idiom, these four source domains share a semantic overlap area representing the fundamental necessities of human life.

A total of 113 cooperative activation idioms, constituting 36.4% in *Developing Chinese*, showcase the versatility of this activation pattern. Examples include “白马王子” (báimǎ wángzǐ, Prince Charming), “鼻青脸肿” (bíqīng liǎnzhuàng, black and blue), “垂头丧气” (chuítóu sàngqì, dejected), featuring common or similar semantic ranges that create an overlapping region directing the idiomatic meaning toward the target domain.

#### 3.1.3 Spreading activation idioms

Single Source Category idioms, falling under the Spreading Activation category, exhibit a specific activation pattern. These idioms, totaling 120 and constituting 38.7% in *Developing Chinese*, have either a single ordinary source domain or a single source domain rooted in folklore. For instance, “鹤立鸡群” (hè lì jī qún) represents a singular source domain. Once activated, it establishes a semantic network with multiple pathways, but only one of these pathways represents the actual meaning of the idiom. According to *The Dictionary of Idioms*, it means “standing out like a crane among chickens,” metaphorically describing someone with exceptional appearance or talent.

These idioms lack directed target domains or cooperative source domains, and their meanings exhibit spreading and uncertainty. In their semantic networks, one particular path, for uncertain reasons, is repeatedly strengthened and highlighted, forming the ultimate meaning. This activation pattern is referred to as Spreading-Activation.

#### 3.1.4 Superimposed activation idioms

Superimposed Activation Idioms, with allegorical status, incorporate a folklore-based source domain alongside an ordinary one. The idiom “塞翁失马” (sàiwēng shīmǎ) exemplifies this category, activating a story from the “Huainanzi” alongside its ordinary source domain. This activation pattern involves two activations, one from idiom to folklore and the other from folklore to meaning, with the folklore serving as a bridge to correctly understand the idiom’s meaning. The story goes as follows:

近塞上之人, 有善术者, 马无故亡而入胡。人皆吊之, 其父曰:“此何遽不为福乎?“居数月, 其马将胡骏马而归。人皆贺之, 其父曰:“此何遽不能为祸乎?“家富良马, 其子好骑, 堕而折其髀。人皆吊之, 其父曰:“此何遽不为福乎?“居一年, 胡人大入塞, 丁壮者引弦而战。近塞之人, 死者十九。此独以跛之故, 父子相保。.

In conclusion, these distinct activation patterns offer unique perspectives on Chinese idioms, revealing intricate relationships between source and target domains, enriching the understanding of these linguistic expressions.

### 3.2 Statistical analysis of idiom categories in *Developing Chinese*

The idiomatic data selected for this study are exclusively sourced from the six-volume series *Developing Chinese*. According to the statistics, a total of 476 idioms are found throughout the six volumes. Among these, 310 idioms belong to the category of metaphorical idioms, accounting for approximately 65.1%, while 166 idioms fall into the non-metaphorical category, constituting approximately 34.9%. It is evident that the majority of the idioms selected in the Chinese language teaching materials fall under the category of metaphorical idioms. Assuming that this selection reflects a representative sampling, the distribution of idioms in actual language materials would likely follow a similar pattern, with a substantial proportion consisting of metaphorical idioms.

Based on the method presented above, the idioms appearing in *Developing Chinese* textbooks (all six volumes) are classified into four categories, as shown in Table 1.

**Table 1:** Statistical analysis of idiom categories in *Developing Chinese.*

Total classes	Subclass	Quantity	Percentage
Metaphorical and metonymic idioms	Directional activation idioms	56	18.1%
	Cooperative activation idioms	113	36.4%
	Spreading activation idioms	120	38.7%
	Superimposed activation idioms	21	6.8%

Among these, there are 56 directional activation idioms, accounting for 18.1%, 113 cooperative activation idioms, making up 36.4%, 120 spreading activation idioms, representing 38.7%, and 21 superimposed activation idioms, constituting 6.8%.

### 3.3 Cognitive difficulty result of four categories of the CM&MIs

This section is primarily concerned with evaluating the complexity of these activation patterns.

Firstly, in terms of activation number, except for superimposed activation, directional activation, cooperative activation, and spreading activation each involve a single activation event. Therefore, these three activation categories are simpler compared to superimposed activation, which necessitates two activation events.

Secondly, concerning the presence of assistance, directional and cooperative activation types benefit from assistance, while spreading and superimposed activation types do not. Consequently, the latter two are more complex in comparison to the former two.

Lastly, we examine the nature of assistance, focusing on directional and cooperative activation. For directional activation, the assistance involves targeting the activating object, namely the source domain, and defining a clear objective. Specifically, this entails establishing a meaningful target for the source domain. After activation, the activation energy is directed toward the target domain, providing a definite direction for activation. In such a scenario, activation is unidirectional and deterministic. In contrast, cooperative activation relies on semantic cooperation. In this mode, different source domains radiate their respective semantic networks, and these distinct semantic networks converge within an overlapping interval, collectively conveying the true meaning of the idiom. Clearly, from the perspective of the nature of assistance, cooperative activation is simpler than directional activation. The summarized analysis is presented in Table 2.

**Table 2:** Difficulty assessment of the four categories of the CM&MIs.

Chinese idioms	Activation number	Assistance required	Nature of assistance
Directional activation	1	+(Yes)	Specify objective
Cooperative activation	1	+(Yes)	Semantic cooperation
Spreading activation	1	-(No)	
Superimposed activation	2	-(No)	

It is important to note that superimposed activation relies on a bridge source domain for its dual activation process. Failure to clearly understand the context of this source domain can lead to misinterpretations, significantly increasing the complexity of this activation pattern.

Based on the above analysis, the varying complexities of different activation patterns are evident. The order from most complex to simplest is as follows: superimposed activation > spreading activation > cooperative activation > directional activation. Correspondingly, the continuum of cognitive difficulty for the four categories of idioms is as follows: idioms based on superimposed activation > idioms based on spreading activation > idioms based on cooperative activation > idioms based on directional activation.

The classification mode of this study and the types of idioms thus divided provide a new research window and path for the study of the CM&MIs, and also provide new theoretical support for the teaching of the CM&MIs.

## 4 Conclusion

This part subsumes a brief summary and the implication for teaching Chinese as a foreign language (TCFL).

### 4.1 A brief summary of CM&MIs

Through the above exploration of the four activation patterns, we have seamlessly integrated these inherent activation modes with the overt characteristics of idioms. These external features serve as clear indicators for distinguishing different idiom categories. As previously mentioned, the source domain is an indispensable component of idioms. In other words, each idiom inherently contains a source domain. By considering the relationship between idioms and their source domains, the number of source domains, and the characteristics of these source domains, it becomes possible to systematically classify existing Chinese idioms in a novel way. In essence, the source domain is an irreplaceable component within the complex structure of idioms. Moreover, at the level of meaning conveyance, the source domain reappears as the object of activation.

Specifically, when both the source domain and the target domain coexist within an idiom, the corresponding activation relationship is directional activation. In such cases, the target domain sets up the semantic target, while the spreading source domain acts as the arrow, highlighting semantically relevant associations with the target domain and hitting the bullseye of the source domain. In this scenario, associative activation within idioms is unidirectional, determinate, and readily discernible. Examples of such idioms include “暴跳如雷” (“explode like thunder”), “受宠若惊” (“be favored and alarmed”), “聪明绝顶” (“extremely clever”), “小心翼翼” (“cautious and meticulous”), “门庭若市” (“crowded with visitors”), and “含情脉脉” (“tender and affectionate”). Once learners establish the relationship between the source domain and the target domain, the iconic characteristics of these idioms facilitate the construction of the idiomatic activation relationship. Consequently, the semantics of these idioms become evident during directional activation.

In cases where only source domains exist, and multiple source domains are involved, the specific activation theory that aligns with this scenario is cooperative activation. In this process, different source domains each radiate various associative meanings, and these meanings intersect in an overlapping interval of consensus meanings. This intersection represents a collective understanding—an activation collision. Together, these consensus meanings cooperatively convey the ultimate meaning of the idiom. Such idioms typically fall into two categories: those with two source domains, such as “鸡毛蒜皮” (“trifles”), “白马王子” (“Prince Charming”), “鼻青脸肿” (“bruised and swollen”), “垂头丧气” (“crestfallen”), and those with four source domains, such as “衣食住行” (“clothing, food, shelter, and transportation”), “油盐酱醋” (“cooking ingredients”), “生老病死” (“birth, aging, illness, and death”), “琴棋书画” (“the four arts: qin, chess, calligraphy, and painting”), “阴晴圆缺” (“waxing and waning”), and “酸甜苦辣” (“the five flavors: sour, sweet, bitter, spicy, and salty”). Idioms of this nature present greater memorization challenges to Chinese learners compared to idioms where the source domain and the target domain coexist. However, the overt characteristic of multiple source domains and the intrinsic cooperative activation mechanism can aid students in memorizing these idioms.

### 4.2 The implication for the TCFL

The SA model significantly aids in understanding idioms, especially when teaching the CM&MIs in Chinese as a foreign language. By applying different activation patterns to classify these idioms, students’ divergent thinking is developed, autonomous learning is promoted, and vocabulary instruction efficiency is enhanced. This approach not only strengthens students’ memory of idioms but also fosters their interest and enthusiasm for proactive vocabulary learning, thereby deepening their understanding of traditional Chinese culture.

Traditional methods for grading idiom difficulty are impractical. The proposed difficulty gradient sorting based on the SA theory offers a more cognitive-friendly approach, benefiting both teaching and textbook arrangement. This sorting follows the cognitive principle of progressing from easy to challenging, improving learning efficiency, and enhancing the instructive and operational qualities of textbooks (Tang, 2010).

## Data availability statement

The raw data supporting the conclusions of this article will be made available by the authors, without undue reservation.

## Author contributions

YX: Methodology, Writing – original draft, Writing – review & editing.
